# Diagnostic accuracy of next-generation sequencing (NGS) for identifying actionable mutations in advanced non-small cell lung cancer: Systematic Review and Meta-Analysis

**DOI:** 10.1007/s12094-025-04040-7

**Published:** 2025-09-13

**Authors:** Nicolás Téllez Castillo, Ana M. Goyeneche-García, Luisa M. Montoya Quesada, Oscar A. Gamboa Garay, Ricardo E. Bruges Maya

**Affiliations:** 1 Faculty of Medicine, Department of Internal Medicine, Pontificia Universidad Javeriana, Bogotá, Colombia; 2 Faculty of Medicine, Department of Clinical Epidemiology and Biostatistics, Pontificia Universidad Javeriana, Bogotá, Colombia; 3 Centro Javeriano Oncología, Hospital San Ignacio, Bogotá, Colombia

**Keywords:** Carcinoma non-small-cell lung, Next-generation sequencing (NGS), Diagnostic accuracy, Actionable mutations, Non-small cell lung cancer (NSCLC), Metanalysis and systematic review

## Abstract

**Purpose:**

To evaluate the diagnostic accuracy and clinical performance of next-generation sequencing (NGS) compared to conventional techniques for detecting actionable mutations using tissue or liquid biopsy samples in patients with advanced non-small cell lung cancer.

**Methods:**

A systematic review and meta-analysis of diagnostic test studies (PROSPERO: CRD42023450465) were conducted. We included studies with sufficient comparative data, using a *t* test to analyze turnaround time differences and hypothesis testing for valid result proportions (*p* < 0.05). The meta-analysis, performed in Stata 17^®^, pooled sensitivities and specificities by mutation and evaluation technique. The QUADAS-2 tool assessed study quality.

**Results:**

A total of 56 studies involving 7143 patients were analyzed. No significant differences were found in valid result percentages between standard tests and NGS in tissue (85.57% vs. 85.78%; p = 0.99) and liquid biopsy (81.50% vs. 91.72%; p = 0.277). Liquid biopsy had a significantly shorter turnaround time (8.18 vs. 19.75 days; p < 0.001). NGS demonstrated high accuracy in tissue for EGFR (sensitivity: 93%, specificity: 97%) and ALK rearrangements (sensitivity: 99%, specificity: 98%). In liquid biopsy, NGS was effective for EGFR, BRAF V600E, KRAS G12C, and HER2 (sensitivity: 80%, specificity: 99%) but had limited sensitivity for ALK, ROS1, RET, and NTRK rearrangements.

**Conclusions:**

NGS enables comprehensive mutation analysis, particularly for point mutations. Further validation is required to improve the detection of gene rearrangements.

**Supplementary Information:**

The online version contains supplementary material available at 10.1007/s12094-025-04040-7.

## Introduction

Lung cancer remains one of the Leading causes of cancer incidence and mortality worldwide, with 2.4 million new cases and 1.8 million associated deaths reported in 2022 [1, 2]. This high disease burden accounts for over 46.5 million disability-adjusted Life years, reflecting an 18.3% increase since 2010 [3]. Advancements in cancer research have facilitated the identification of actionable mutations (AM), which predict responses to specific treatments and have shifted the treatment paradigm towards precision medicine [4–7]. Current guidelines recommend identifying AM in all patients with advanced non-small cell lung cancer (NSCLC), either sequentially or concurrently [8–10], facilitating the delivery of targeted therapies with significant clinical benefits.

Tissue samples remain the standard for tumor profiling; therefore, preserving tissue is a priority to ensure adequate material for current and future studies. As an alternative, circulating tumor DNA (ctDNA) has been proposed as a real-time, noninvasive biomarker, providing prognostic and predictive information for treatment monitoring and enabling mutation detection when tissue is unavailable due to its high concordance with tissue-based analyses [11–13]. However, the detection of ctDNA has limitations, including interference from clonal hematopoiesis, which can result in false positives and misguided treatment recommendations [11, 14]. Its sensitivity also correlates with tumor burden, which is generally higher in patients with advanced disease [15]. These limitations increase the risk of invalid results, including artifacts and mutations for which no therapeutic options are available [16].

To maximize the use of available samples from tumor tissue (TT) and liquid biopsy (LB), techniques that identify the highest number of alterations while ensuring the rational use of resources should be prioritized [8]. Next-generation sequencing (NGS) is the recommended approach for simultaneously evaluating mutations, facilitating the early initiation of personalized therapy [8, 17]. Advances in technology have made these analyses more accessible for clinical use.

This systematic literature review and meta-analysis aim to provide information on the diagnostic performance of NGS compared to other techniques for identifying AM in TT samples or LB samples from patients diagnosed with NSCLC who are eligible for targeted therapy.

## Material and methods

### Study identification and study selection

A systematic Literature search was conducted from September to October 2023 in the PubMed and EMBASE databases. The search was not restricted by publication date or language. Studies were included if they involved patients with advanced NSCLC, defined as locally advanced (stage III) or disseminated disease (stage IV) according to the American Joint Committee on Cancer (AJCC) staging system, and reported the evaluation of AM using NGS compared to another diagnostic technique. The detailed search strategy and terms used for each database were previously specified in the published protocol [18] and are summarized in Supplementary Table 1.

Inclusion criteria were confirmed NSCLC diagnosis based on cytological or histopathological evaluation, classified as advanced stage; Paired tissue and/or blood samples obtained from the same patient; Detection of AM using standard diagnostic techniques; Specification of the test performed for each sample type (tissue or plasma); and availability of sufficient data to construct a 2 × 2 diagnostic contingency table. Exclusion criteria were: Use of cell lines or artificial samples; Inability to extract relevant data; Non-NSCLC histology; and Lack of TT for AM testing.

Two investigators (NT and LV) independently screened the studies using the Rayyan virtual platform [18], performing blinded reviews of titles and abstracts. Discrepancies were resolved through discussion, and if consensus could not be reached, a third reviewer (AG) acted as arbiter to determine final eligibility for full-text assessment.

For studies meeting the inclusion criteria but lacking complete data, a letter was sent to the corresponding author requesting the missing information or raw data for manual extraction. Conference abstracts were reviewed to verify the original article's publication and potential inclusion. A snowballing strategy was employed to identify additional relevant articles [19–22], selecting those that met the inclusion criteria but were not identified in the initial search.

### Data extraction and data analysis

Two investigators (NT and AG) jointly extracted data from the selected studies and stored the information in Microsoft Excel^®^. For each study, we recorded the number of samples analyzed using the standard method and the NGS intervention, along with the sample type (tissue or plasma) and the sequencing platform used. For each mutation, we extracted the total number of samples analyzed, true positives, false positives, false negatives, and true negatives. Data were obtained from the original publication, supplementary materials, or through direct communication with the corresponding authors. Only studies that reported—or provided sufficient information to calculate—sensitivity and specificity for at least one actionable mutation were included. When these metrics were not explicitly stated, they were calculated manually using raw data from the article, supplementary files, or information provided by the authors.

Standard-of-care (SOC) techniques were defined as those recommended by current clinical guidelines for routine mutation analysis. For the detection of point mutations, SOC methods included PCR-based assays. For gene rearrangements, initial screening was typically performed using immunohistochemistry (IHC), with confirmatory testing through fluorescence in situ hybridization (FISH) or other in situ hybridization (ISH) methods, as per standard diagnostic algorithms. If the standard method was not explicitly described, it was assumed to be tissue-based testing. The specific biomarkers assessed across all included studies are summarized in Supplementary Table 2.

For both the standard method and the intervention, the analysis included, whenever available, the number of valid tests, defined as the number of processed samples that yielded an interpretable result (either positive or negative) out of the total analyzed samples. Turnaround time (TAT) was also recorded as the time from sample collection to obtaining a valid result [23].

To explore the potential clinical utility of different NGS approaches, we included the distinction between commercial and in-house NGS panels. A commercial NGS panel was defined as a pre-designed sequencing kit developed by specialized laboratories, typically validated for clinical use and widely available for purchase. In contrast, an in-house NGS panel refers to a customized assay developed internally by a diagnostic or research laboratory, usually targeting a selected set of genomic regions based on institutional needs or local prevalence patterns. Evaluating whether there are significant differences in diagnostic performance between both types of panels is essential to determining their clinical reliability and scalability. This distinction is particularly relevant for institutions with limited access to commercial platforms, as demonstrating comparable accuracy would support the use of validated in-house panels as a feasible and cost-effective alternative in routine practice.

A hypothesis test for proportion differences was performed to compare the percentage of valid results between sample types (TT vs. TT and TT vs. LB), considering a *p* value < 0.05 as statistically significant. To assess differences in TAT between the standard method and the comparator, a t-test for mean differences was conducted, with statistical significance set at *p* < 0.05. All analyses were performed using Stata 17^®^.

### Quality assessment of the evidence

The quality of the evidence was assessed using the Quality Assessment of Diagnostic Accuracy Studies 2 (QUADAS-2) tool [24]. To ensure accuracy, the evaluation was conducted in pairs (LI, SG, GN, CT, MC). A risk of bias and applicability table was constructed, and the results were presented graphically. Any discrepancies were resolved by consensus among the reviewers.

### Meta-analysis

The following exclusion criteria were applied for the meta-analysis: (1) Studies that did not adequately specify the type of evaluation performed on the sample; (2) Studies that used multiple tests for mutation assessment; (3) Studies where sensitivity and specificity calculations were not feasible; (4) Studies that did not meet the minimum requirement of four studies conducting a direct evaluation of these mutations.

For each diagnostic test evaluated, data from relevant studies were synthesized and summarized, including reported sensitivity and specificity values. When multiple studies were available for a given test and provided sufficient data on true positives, false positives, true negatives, and false negatives, the feasibility of calculating a pooled estimate through meta-analysis was assessed.

Statistical analysis was performed using Stata 17^®^, applying the "metadta" command for each mutation, evaluation test, and reference standard. This function enabled a meta-analysis of diagnostic tests, pooling the reported sensitivities and specificities from the included studies. Additionally, forest plots were generated to visualize individual and combined estimates across studies.

Statistical heterogeneity was not assessed using the *I*^2^ statistic commonly applied in conventional meta-analyses, as it evaluates only a single outcome at a time and does not account for the threshold effect. Given the potential correlation between sensitivity and specificity, *I*^2^ was deemed unsuitable for this context [25]. Instead, heterogeneity was assessed by examining the variability of study results in the sensitivity and specificity forest plots.

The protocol for this review was previously registered in PROSPERO under the code CRD42023450465 and has been published [18].

## Results

**Fig. 1:**
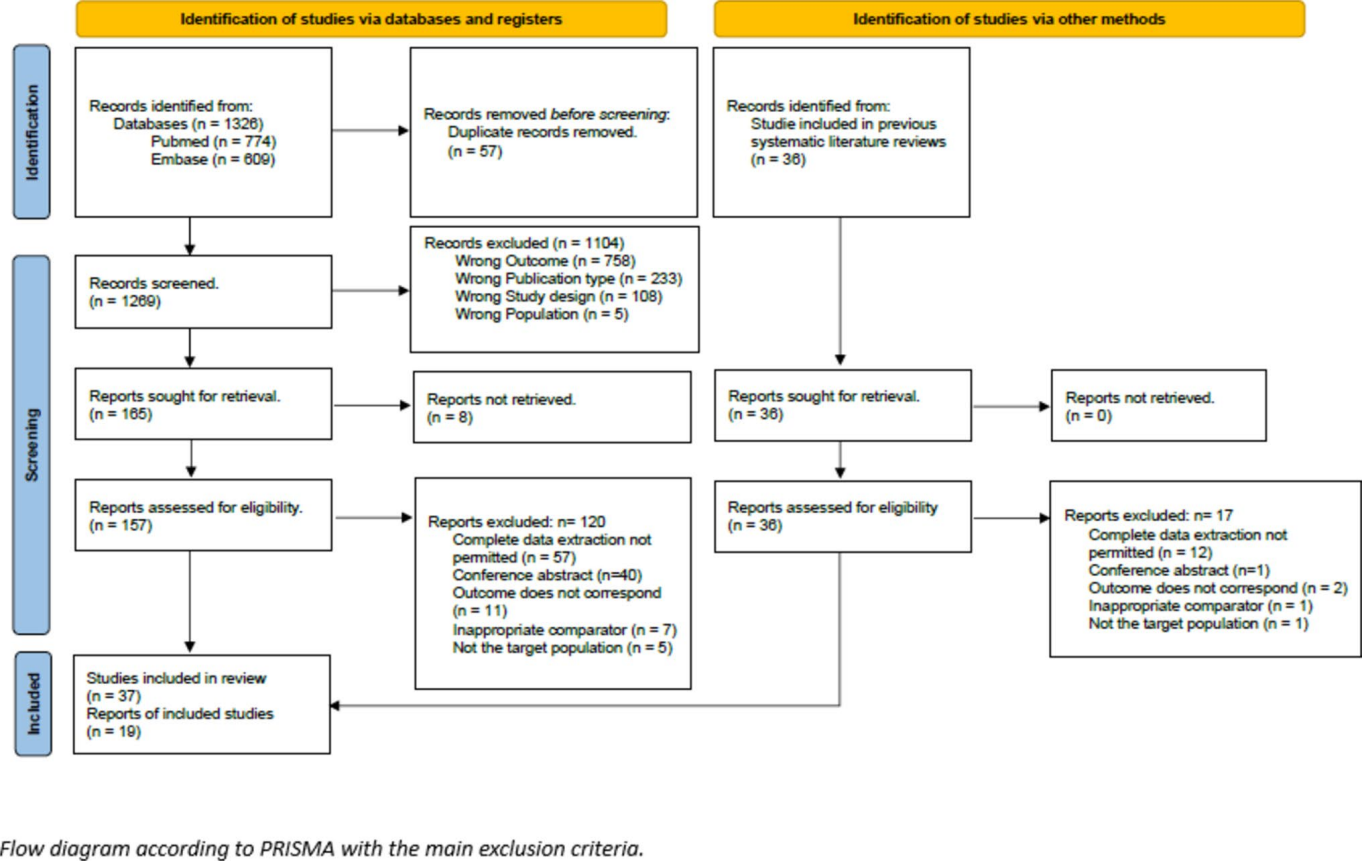
PRISMA flow diagram

The search was concluded in September 2023, identifying 1269 articles after removing duplicates. A total of 56 articles were included in the analysis [26–81] following the application of inclusion and exclusion criteria (Fig. 1). Table 1 summarizes the evaluations conducted, specifying each standard method and comparison. In Evaluation 1, NGS results in tissue were compared with standard PCR methods in tissue and IHC/FISH for rearrangements. In Evaluation 2, the accuracy of NGS in LB was assessed in comparison with standard methods in tissue, including NGS. Finally, in Evaluation 3, the differences in diagnostic performance between commercial and in-house NGS panels in LB were analyzed, comparing them with standard methods in tissue. Patient characteristics for the included studies are detailed in Supplementary Table 3.

**Table 1:** Standard methods and comparisons for evaluations

	Standard method	Comparison method
Evaluation 1	PCR methods in tissue	NGS methods in tissue
	IHC + FISH in tissue	
Evaluation 2	PCR methods in tissue	NGS methods in liquid biopsy
	IHC + FISH in tissue	
	NGS methods in tissue	
Evaluation 3	PCR methods in tissue	NGS commercial methods in liquid biopsy NGS in house methods in liquid biopsy
	IHC + FISH in tissue	
	NGS methods in tissue	

For standard studies, those in which mutation detection was performed using PCR methods in tissue to evaluate mutations in EGFR, KRAS G12C, BRAF V600E, MET Exon 14 Skipping, and HER2 Exon 20 were selected. The identification of rearrangements in ALK, ROS1, RET, NTRK and focal amplifications of MET was carried out in tissue using immunohistochemistry with confirmation by FISH. Fluorescence In Situ Hybridization (FISH), Immunohistochemistry (IHC), Next-Generation Sequencing (NGS), Polymerase Chain Reaction (PCR)

### Percentage of valid results and turnaround time

Seven comparisons reported the proportion of valid results for standard tissue-based tests, with a mean of 85.57% (95% confidence interval [CI] 59.54–111.60%). Nine comparisons reported valid results for NGS in tissue, with a mean of 85.78% (95% CI 62.96–108.60%). No statistically significant difference was found between both methods (p = 0.99) (Supplementary Figure 1). TAT was not reported for the tissue-based test comparisons.

In tissue-based tests, including standard tests and NGS, 22 comparisons reported valid results, with a mean of 81.50% (95% CI 65.27–97.73%). In LB-based NGS tests, 29 comparisons reported valid results, with a mean of 91.72% (95% CI 81.70–101.75%). No statistically significant difference was found between both methods (p = 0.277) (Supplementary Figure 2).

Four comparisons reported the TAT for tissue-based tests, with a mean of 19.75 days (95% CI 14.49–25 days). The weighted mean TAT reported in 11 comparisons for LB-based tests was 8.18 days (95% CI 6.19–10.17 days). The mean difference between both methods was − 11.57 days (95% CI − 15.41 to − 7.73 days), indicating that LB tests provide significantly faster results than tissue-based tests (p < 0.001) (Supplementary Figure 3).

### Evaluation of SOC in tissue compared to NGS in tissue (evaluation 1)

Twelve studies with 15 comparisons assessed the performance of NGS in TT or cytology, comparing it to standard tests. For the meta-analysis, 13 comparisons were selected for two genes: EGFR (9 comparisons) and ALK (4 comparisons). The overall sensitivity for detecting EGFR mutations via NGS in tissue was 93% (95% CI 88–96%), with a specificity of 97% (95% CI 91–99%). The overall sensitivity for detecting ALK rearrangements was 99% (95% CI 16–100%), with a specificity of 98% (95% CI 88–100%) (Table 2, Supplementary Figures 4 and 5). Insufficient comparisons were available to perform a meta-analysis of tissue-based NGS for other mutations.

**Table 2:** Performance of NGS in tissue compared to SOC in tissue

Gene	Eval	Sen (%)	CI—95%	Spe (%)	CI—95%
EGFR	PCR TT NGS TT	93	88–96	97	91–99
ALK	IHC + FISH NGS TT	99	16–100	98	88–100

Diagnostic Performance of NGS in tissue compared to SOC in tissue. Anaplastic Lymphoma Kinase (ALK), Confidence Interval (CI), Evaluation (Eval), Epidermal Growth Factor Receptor (EGFR), Fluorescence In Situ Hybridization (FISH), Immunohistochemistry (IHC), Next-Generation Sequencing (NGS), Polymerase Chain Reaction (PCR), Sensitivity (Sen), Specificity (Spe), Standard of Care (SOC), Tumor Tissue (TT)

### Performance of NGS in liquid biopsy for mutation and rearrangement detection (evaluation 2)

We assessed the performance of NGS in LB for detecting EGFR alterations, KRAS G12C mutation, BRAF V600E mutation, and HER2 exon 20 alterations by analyzing 63 comparisons between standard tests and NGS in TT. The overall sensitivity for detecting these alterations was 80% (95% CI 75–84%), with a specificity of 99% (95% CI 96–100%) (Supplementary Figure 6).

In 32 comparisons, the performance of NGS in LB was evaluated for detecting rearrangements in ALK, RET, ROS1, and NTRK, compared with standard tests and NGS in TT. The overall sensitivity for detecting these rearrangements was 66% (95% CI 53–76%), with a specificity of 100% (95% CI 99–100%) (Supplementary Figure 7) (Table 3).

**Table 3:** Performance of NGS in liquid biopsy compared to SOC + NGS in tissue

Gene	Sen (%)	CI—95%	Spe (%)	CI—95%
KRAS G12C	82	66–91	100	98–100
EGFR	80	75–84	97	94–99
BRAF V600E	78	53–91	100	99–100
ALK	69	57–78	100	99–100
MET	57	42–70	100	98–100
RET	53	32–72	100	99–100
ROS1	30	12–56	100	98–100

Diagnostic Performance of NGS in Liquid Biopsy Compared to SOC Including NGS in tissue. Anaplastic Lymphoma Kinase (ALK), B-Raf Proto-Oncogene, V600E mutation (BRAF V600E), Epidermal Growth Factor Receptor (EGFR), Kirsten Rat Sarcoma Viral Oncogene Homolog (KRAS), Mesenchymal-Epithelial Transition factor (MET), Next-generation sequencing (NGS), Rearranged during Transfection (RET), c-ros Oncogene 1 (ROS1), Sensitivity (Sen), Specificity (Spe), Standard of care (SOC), Confidence Interval (CI)

Across 44 studies, 51 comparisons were made between standard tests and NGS in TT versus NGS in LB for seven genes: EGFR, KRAS, BRAF, ALK, MET, RET, and ROS1 (Table 4). The forest plots for each gene are shown in Supplementary Figures 8–14, and Supplementary Table 4 presents the results based on the type of standard test evaluated.

**Table 4:** Performance of commercial and in-house NGS panels in liquid biopsy for detection of EGFR mutations Compared to PCR or NGS in tissue

	Sen. (CI-95%)	*P*		Spe. (CI-95%)	*P*
*PCR in tissue versus NGS Liquid biopsy*					
Commercial	86.3% (71.5–94.0%)	0.606	Commercial	94.3% (72.48–99.0%)	0.668
In-House	74.7% (65.5–82.1%)		In-House	99.3% (93.89–99.9%)	
*NGS in tissue versus NGS Liquid biopsy*					
Commercial	77.8% (69.8–84.0%)	0.647	Commercial	96.2% (91.0–98.5%)	0.935
In-House	87.0% (79.6—92.0%)		In-House	97.3% (77.9–99.7%)	

Diagnostic Performance of Commercial and In-House NGS Panels in Liquid Biopsy Compared to Standard Tissue Studies, Including NGS, for the Detection of EGFR Mutations. Confidence Interval (CI), Epidermal Growth Factor Receptor (EGFR), Next-generation sequencing (NGS), Polymerase Chain Reaction (PCR), Sensitivity (Sen), Specificity (Spe)

There were not enough comparisons to perform a meta-analysis of NGS in tissue for NTRK or HER2 mutations. Two studies [76, 78] evaluated the diagnostic performance of NTRK detection in LB. The detection method in tissue was NGS, and sensitivity and specificity exceeded 99% for identifying this fusion in LB.

Eight studies [28, 29, 36, 50, 52–54, 59] assessed HER2 exon 20 mutations using NGS in tissue. However, diagnostic performance could only be calculated in seven studies, showing high variability in sensitivity, ranging from 50 to 100%, while specificity ranged from 95 to 100%.

### Diagnostic performance of NGS in LB using commercial or in-house panels compared to SOC + NGS in tissue (evaluation 3)

Thirty-six comparisons were analyzed, evaluating commercial and in-house NGS panels in LB and comparing their results with standard tests or NGS in tissue. Sufficient data were available to assess the diagnostic performance of commercial and in-house panels for detecting EGFR mutations (Table 4).

In eight comparisons, PCR in tissue was used as the standard test. The commercial NGS panel in LB demonstrated a sensitivity of 86.2% (95% CI 71.4–94.0%) and a specificity of 94.2% (95% CI 72.4–99.0%). The in-house NGS panel in LB had a sensitivity of 74.7% (95% CI 65.4–82.1%) and a specificity of 99.4% (95% CI 93.8–99.9%). No statistically significant differences were found between the two panels in sensitivity (p = 0.606) or specificity (p = 0.668).

Similar results were observed when comparing NGS in tissue versus NGS in LB. The commercial NGS panel in LB showed a sensitivity of 77.7% (95% CI 69.7–84.0%) and a specificity of 96.2% (95% CI 91.0–98.5%). The in-house NGS panel in LB demonstrated a sensitivity of 87.1% (95% CI 79.6–92.0%) and a specificity of 97.3% (95% CI 77.9–99.7%). Again, no significant differences between the two panels in sensitivity (p = 0.647) or specificity (p = 0.935) were found. Insufficient comparisons were available to assess the diagnostic performance of in-house panels for detecting other mutations.

### Quality of the evidence

**Fig. 2:**
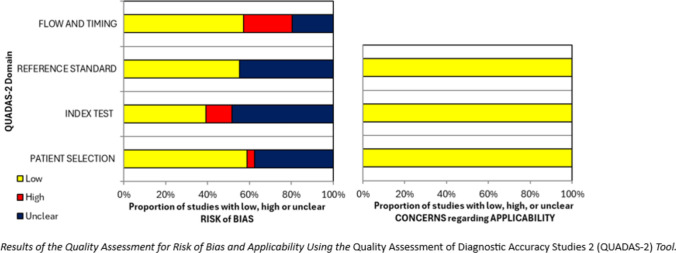
Summary of QUADAS-2 quality assessment

The quality of evidence is detailed in Supplementary Tables 5 and 6 and illustrated in Fig. 2. All studies showed a low risk in the applicability domains. Regarding the risk of bias: In the patient selection domain, 59% of studies had a low risk, 4% had a high risk due to the inclusion of non-randomized patients or inappropriate criteria, and 20% lacked sufficient information. In the index test domain, 39% of studies had a low risk of bias, 13% had a high risk due to awareness of standard test results, and 48% lacked sufficient information. In the reference standard domain, 55% of studies had a low risk of bias, while 45% lacked sufficient information. In the flow and timing domain, 57% of studies had a low risk, 23% had a high risk due to inappropriate time intervals, and 20% lacked sufficient information.

## Discussion

Precision oncology approaches based on comprehensive genomic characterization have become the standard of care in patients with advanced NSCLC, targeted therapies have demonstrated significant improvements in overall survival for those harboring AM [82–84]. Real-world data highlight critical gaps in the implementation of molecular testing. In the first-line treatment setting, approximately 24% of patients with de novo metastatic NSCLC had no biomarker results available for any guideline-recommended AM or PD-L1 at treatment initiation. Among those tested, only 19% had results for all four key AM (ALK, EGFR, ROS1, BRAF) prior to starting first-line therapy, underscoring the suboptimal uptake of comprehensive molecular profiling in routine clinical practice [85]. This substantial gap limits patient access to targeted therapies and has been consistently associated with delayed therapeutic initiation, increased reliance on non-targeted systemic treatments, and worse survival outcomes compared to patients receiving genotype-guided therapy [86]. NGS has emerged as a promising tool for identifying AM in advanced NSCLC, enabling the simultaneous assessment of multiple genetic mutations and LB as a critical component in providing minimally invasive molecular profiling, especially when tissue samples are insufficient, which can help optimize patient management.

However, despite its potential to guide effective treatment, the diagnostic TAT remains a crucial factor. Prolonged time to treatment has been associated with poorer outcomes in NSCLC, even after adjusting for stage at presentation [87–89]. Recognizing this, several international oncology societies have proposed acceptable diagnostic-to-treatment intervals ranging from 30 to 52 days [87, 90]. In our study, LB-based tests were associated with a significantly shorter median TAT (8.18 days) compared to tissue-based testing (19.75 days), without statistically significant differences in the proportion of valid results between tissue and LB approaches. This suggests that LB can expedite molecular diagnosis and enable earlier treatment initiation without compromising diagnostic reliability. Faster TAT can help reduce the time to optimal treatment decision facilitating initiation of targeted therapies, which may ultimately improve clinical outcomes [91]. When evaluating the diagnostic performance of NGS in tissue compared to standard tests, our results demonstrated high sensitivity and specificity, particularly for detecting the most frequent mutations in NSCLC, EGFR alterations, and ALK rearrangements [92]. This finding is crucial, as it supports the feasibility of accurate mutation detection, reducing the need for sequential testing and minimizing tumor sample depletion [93]. The performance of NGS for detecting AM in LB, NGS in peripheral blood showed adequate sensitivity for detecting EGFR alterations, KRAS G12C mutations, and BRAF V600E mutations, with high specificity. However, NGS demonstrated lower sensitivity for detecting gene rearrangements in ALK, ROS1, and RET. This may be attributed to the nature of these alterations, which often require complementary techniques like FISH or IHC for accurate detection. Although the number of comparisons evaluating NTRK rearrangements was insufficient, our results showed high sensitivity and specificity for NGS-based LB in this setting, contrasting with findings for other rearrangements. Additional comparative studies are needed to confirm this observation.

Our findings are consistent with the literature, which highlights the limitations of NGS in capturing complex structural variants, particularly when using DNA-based sequencing [94, 95]. DNA-based NGS can be constrained in identifying gene fusions due to large intronic regions that hinder amplification and sequencing [96, 97]. We did not differentiate between DNA- and RNA-based NGS for rearrangement detection, which limits the ability to evaluate each technique separately; therefore, these results should be interpreted with caution. RNA-based NGS evaluates mature RNA Transcripts and is unaffected by intron size, enabling more reliable detection of gene fusions. Notably, RNA sequencing has demonstrated higher sensitivity for identifying MET exon 14 skipping events compared to DNA-based approaches and can also outperform DNA-based testing in detecting point mutations, insertions, or deletions that alter splicing and transcript structure [97, 98].

We evaluated whether diagnostic performance differed between commercial and in-house NGS panels in LB. The analysis was limited to EGFR mutations, revealing no statistically significant differences in diagnostic accuracy for detecting AM. In-house sequencing panels may provide a cost-effective alternative and could help reduce TAT; however, they require rigorous internal and external validation to ensure data reliability [23]. Although further studies are needed to confirm these findings for other mutations, in-house panels may be a viable cost-reduction strategy.

Our review allowed for the individual assessment of each mutation, determining the specific performance of NGS. We included NTRK rearrangements, and HER2 exon 20 mutations recommended for targeted treatment in patients with advanced NSCLC. To ensure a focused and clinically meaningful evaluation, we limited our analysis to selected alterations with well-defined therapeutic implications—specifically KRAS G12C and BRAF V600E—while excluding other mutations with limited clinical evidence. We also incorporated the analysis of clinical variables, including TAT and the proportion of valid results, to evaluate NGS's operational feasibility and diagnostic reliability in both TT and LB.

Despite efforts to contact authors, a high exclusion rate due to missing data limited our ability to analyze low-frequency genes. Furthermore, clonal hematopoiesis can interfere with ctDNA analysis, particularly when the allelic fraction in blood is not accounted for. This can potentially lead to the detection of non-cancerous mutations and increase the risk of false positives, thus impacting test accuracy.

Additional studies are needed to enhance NGS's sensitivity for detecting gene rearrangements in LB and explore its impact on clinical outcomes. Furthermore, standardizing the validation of commercial and in-house panels will be essential for improving result reproducibility. Prospective studies linking NGS findings with treatment response and survival outcomes will further define its role in precision oncology.

## Conclusions

Our systematic review and meta-analysis evaluated the diagnostic performance of NGS for detecting actionable mutations in NSCLC and provided key insights into its clinical applicability in both TT and LB. This approach demonstrated significantly shorter TAT in LB while maintaining high concordance with standard tissue-based methods. NGS techniques showed high sensitivity and specificity for AM in NSCLC but had limitations in detecting gene rearrangements. Clonal hematopoiesis may lead to false positives and compromise test accuracy, and the detection of low-frequency variants in LB remains technically challenging, especially in patients with low tumor burden, potentially affecting overall sensitivity.

Further validation and mitigation strategies are needed to address these issues, with emphasis on improving rearrangement detection through RNA-based sequencing approaches. In-house NGS panels may offer reduced TAT and a cost-effective alternative to commercial assays; however, their clinical implementation requires rigorous internal and external validation, and current evidence is limited. By enabling the simultaneous assessment of multiple mutations in a single sample, NGS facilitates personalized therapy. Its high concordance with standard methods and applicability in LB support its integration into clinical practice, particularly for patients with limited tissue, minimizing the need for repeat biopsies and enhancing precision oncology.

## Supplementary Information

Below is the link to the electronic supplementary material.

Supplementary file1 (DOCX 1108 KB)

## Data Availability

Not applicable.
