# Supplementary material for: Diagnostic accuracy of next-generation sequencing (NGS) for identifying actionable mutations in advanced non-small cell lung cancer: Systematic Review and Meta-Analysis
Source: Clin Transl Oncol. 2025 Sep 13;28(3):1005–15. doi: 10.1007/s12094-025-04040-7 (PMC12920734; doi:10.1007/s12094-025-04040-7)
Supplement: Supplementary file 1 — Supplementary file1 (DOCX 1108 KB) [file 12094_2025_4040_MOESM1_ESM.docx]

***Supplementary Table 1 – Mesh and EMTREE terms***

***MEDLINE-PubMed***

| Line | Parameter | Terms | No hits |
| --- | --- | --- | --- |
| 1 | Population | ("Adult"[Mesh]) AND "Carcinoma, Non-Small-Cell Lung"[Mesh] | 33.153 |
| 2 | Intervention | "High-Throughput Nucleotide Sequencing"[Mesh] OR “NGS” OR Next-generation sequencing | 102.120 |
| 3 | Outcome | (sensitiv*[Title/Abstract] OR sensitivity and specificity[MeSH Terms] OR diagnose[Title/Abstract] OR diagnosed[Title/Abstract] OR diagnoses[Title/Abstract] OR diagnosing[Title/Abstract] OR diagnosis[Title/Abstract] OR diagnostic[Title/Abstract] OR diagnosis[MeSH:noexp] OR (diagnostic equipment[MeSH:noexp] OR diagnostic errors[MeSH:noexp] OR diagnostic imaging[MeSH:noexp] OR diagnostic services[MeSH:noexp]) OR diagnosis, differential[MeSH:noexp] OR diagnosis[Subheading:noexp]) | 6.491.527 |

***EMBASE***

| Line | Parameter | Terms | No hits |
| --- | --- | --- | --- |
| 1 | Population | ('non small cell lung cancer'/exp OR 'bronchial non small cell cancer' OR 'bronchial non small cell carcinoma' OR 'carcinoma, non-small-cell lung' OR 'lung cancer, non small cell' OR 'lung non small cell cancer' OR 'lung non small cell carcinoma' OR 'non oat cell lung cancer' OR 'non small cell bronchial cancer' OR 'non small cell cancer, lung' OR 'non small cell lung cancer' OR 'non small cell lung carcinoma' OR 'non small cell pulmonary cancer' OR 'non small cell pulmonary carcinoma' OR 'non squamous nsclc' OR 'non-oat cell lung cancer' OR 'non-small-cell lung carcinoma' OR 'nonsmall cell carcinoma of the lung' OR 'nonsmall cell lung cancer' OR 'nonsmall cell lung carcinoma' OR 'pulmonary non small cell cancer' OR 'pulmonary non small cell carcinoma') | 226.450 |
| 3 | Intervention | ('high throughput sequencing'/exp OR 'ngs analysis (next generation sequence analysis)' OR 'high through-put nucleotide sequencing' OR 'high through-put sequence analysis' OR 'high through-put sequencing' OR 'high throughput nucleotide sequence analysis' OR 'high throughput nucleotide sequencing' OR 'high throughput sequence analysis' OR 'high throughput sequencing' OR 'high-throughput nucleotide sequencing' OR 'next generation sequence analysis' OR 'next generation sequencing' OR 'next generation sequencing technology' OR 'next-gen sequence analysis' OR 'next-gen sequencing') | 193.706 |
| 5 | Outcome | ('diagnostic procedure'/de OR diagnosis/de OR 'diagnostic accuracy'/de) | 1.834.813 |

***Supplementary Table 2 – Actionable mutations included***

| Gene | Mutation |
| --- | --- |
| **EGFR** | Exon 19 deletion |
|  | L858R exon 21 |
|  | T790M exon 20 |
|  | G719X exon 18 |
|  | L861Q exon 21 |
|  | S768I exon 20 |
|  | Exon 20 insertions |
| **ALK** | Rearrangements |
| **ROS1** | Rearrangements |
| **NTRK** | Rearrangements |
| **RET** | Rearrangements |
| **MET** | Exon 14 skipping mutation |
|  | Focal amplifications |
| **BRAF** | V600E Mutation |
| **KRAS** | G12C Mutation |
| **HER2/ERBB2** | Exon 20 alterations |

*Anaplastic Lymphoma Kinase (ALK), B-Raf Proto-Oncogene (BRAF), Epidermal Growth Factor Receptor (EGFR), Human Epidermal Growth Factor Receptor 2 (HER2/ERBB2), Kirsten Rat Sarcoma Viral Oncogene Homolog (KRAS), MET Proto-Oncogene (MET), Neurotrophic Tyrosine Receptor Kinase (NTRK), Ret Proto-Oncogene (RET), C-Ros Proto-Oncogene 1 (ROS1).*

***Supplementary Table 3 – Clinical characteristics***

| Type of study | (n) | (%) |
| --- | --- | --- |
| *Prospective* | 44 | 78.57% |
| *Retrospective* | 10 | 17.86% |
| *Prospective and retrospective* | 2 | 3.57% |
|  |  |  |
| Patients |  |  |
| *Total population* | 7143 | 100% |
| *Men* | 3712 | 51.97% |
| *Women* | 3104 | 43.46% |
|  |  |  |
| Smoking status |  |  |
| *No smokers* | 1767 | 24.74% |
| *Smokers* | 3449 | 48.29% |
| *No record* | 172 | 2.41% |
|  |  |  |
| Clinical Stage |  |  |
| *Stage III* | 668 | 9.35% |
| *Stage IV* | 4694 | 65.71% |
| *No record* | 37 | 0.52% |
|  |  |  |
| Histological Characteristics |  |  |
| *Adenocarcinoma* | 5373 | 75.22% |
| *Squamous cell carcinoma* | 542 | 7.59% |
| *Other* | 358 | 5.01% |
| *No record* | 22 | 0.31% |

*Characteristics of patients with advanced NSCLC for whom data were available.*

***Supplementary Table 4 - Performance of NGS in liquid biopsy Compared to SOC + NGS in Tissue***

| ***Gene*** | ***Eval.*** | ***Sen. (%)*** | ***CI - 95%*** | ***Spe. (%)*** | | ***CI - 95%*** | |  |
| --- | --- | --- | --- | --- | --- | --- | --- | --- |
| **KRAS G12C** | NGS TT  NGS LB | 87% | 68-95% | 100% | | 97-100% | |  |
| **EGFR** | PCR TT  NGS LB | 86% | 79-91% | 97% | | 84-99% | |  |
| **BRAF V600E** | NGS TT  NGS LB | 78% | 53-91% | 100% | | 99-100% | |  |
| **EGFR** | NGS TT  NGS LB | 76% | 70-81% | 97% | | 93-99% | |  |
| **ALK** | IHC + FISH  NGS LB | 76% | 50% - 90% | | 100% | | 100% | |
| **ALK** | NGS TT  NGS LB | 66% | 52-78% | | 100% | | 99-100% | |
| **MET** | NGS TT  NGS LB | 57% | 42-70% | | 100% | | 98-100% | |
| **ROS1** | NGS TT  NGS LB | 30% | 12-56% | | 100% | | 98-100% | |

*Anaplastic Lymphoma Kinase (ALK), B-Raf Proto-Oncogene, V600E mutation (BRAF V600E), Confidence Interval (CI), Epidermal Growth Factor Receptor (EGFR), Evaluation (Eval), Fluorescence In Situ Hybridization (FISH), Immunohistochemistry (IHC), Kirsten Rat Sarcoma Viral Oncogene Homolog, G12C mutation (KRAS G12C), Liquid biopsy (LB), MET Proto-Oncogene (MET), Next-Generation Sequencing (NGS), Polymerase Chain Reaction (PCR), C-Ros Proto-Oncogene 1 (ROS1), Sensitivity (Sen), Specificity (Spe), Standard of Care (SOC), Tumor Tissue (TT).*

***Supplementary Table 5 – Risk of Bias Assessment***

| **Risk of bias** | | | | | |
| --- | --- | --- | --- | --- | --- |
| Id |  | **PATIENT SELECTION** | **INDEX TEST** | **REFERENCE STANDARD** | **FLOW AND TIMING** |
| **1** | Leest_2023 | **Low** | **Unclear** | **Low** | **Low** |
| **4** | Murakami_2022 | **Low** | **Low** | **Low** | **High** |
| **5** | Sugimoto_2023 | **Low** | **Low** | **Low** | **Low** |
| **7** | Cui_2022 | **Low** | **Low** | **Unclear** | **Low** |
| **9** | Palmero_2021 | **Low** | **Unclear** | **Unclear** | **Low** |
| **13** | Sepúlveda-Hermosilla_2021 | **Low** | **Unclear** | **Unclear** | **High** |
| **14** | Zhang_2021 | **High** | **Unclear** | **Unclear** | **Low** |
| **23** | Tran_2019 | **Unclear** | **Unclear** | **Low** | **Low** |
| **29** | Dono_2019 | **Unclear** | **Unclear** | **Unclear** | **Low** |
| **30** | Lin_2019 | **Low** | **Unclear** | **Low** | **High** |
| **31** | Li_2019 | **Low** | **Low** | **Low** | **Low** |
| **39** | Malapelle_2017 | **Unclear** | **High** | **Low** | **Low** |
| **40** | Lambros_2017 | **Low** | **High** | **Low** | **Low** |
| **48** | Mehta_2020 | **Low** | **Low** | **Low** | **Low** |
| **50** | Sabari_2019 | **Low** | **High** | **Low** | **High** |
| **58** | Tachon_2019 | **Low** | **Low** | **Low** | **Low** |
| **62** | Lettig_2019 | **Low** | **High** | **Low** | **Unclear** |
| **65** | Francaviglia_2019 | **Unclear** | **Low** | **Low** | **High** |
| **70** | Müller_2017 | **Low** | **Unclear** | **Unclear** | **High** |
| **91** | Reckamp_2016 | **Low** | **Low** | **Low** | **Low** |
| **93** | Paweletz_2016 | **Unclear** | **Low** | **Low** | **High** |
| **96** | Papadopoulou_2019 | **Unclear** | **Unclear** | **Low** | **Unclear** |
| **97** | Rachiglio_2016 | **Unclear** | **Unclear** | **Unclear** | **Low** |
| **98** | Wang_2016 | **Unclear** | **High** | **Low** | **Low** |
| **102** | Couraud_2014 | **Low** | **Unclear** | **Low** | **Low** |
| **104** | Leighl_2019 | **Low** | **Unclear** | **Unclear** | **High** |
| **105** | Remon_2019 | **Low** | **Low** | **Low** | **Low** |
| **106** | Pritchett_2019 | **Low** | **Low** | **Low** | **Low** |
| **108** | Mehta_2021 | **Low** | **Low** | **Low** | **Low** |
| **111** | Lam_2021 | **Unclear** | **Low** | **Low** | **Unclear** |
| **112** | Bauml_2022 | **Low** | **Low** | **Low** | **Low** |
| **113** | Cui_2017 | **Low** | **Unclear** | **Unclear** | **Unclear** |
| **114** | Veldore_2018 | **Unclear** | **Unclear** | **Unclear** | **Low** |
| **115** | Park_2021 | **Unclear** | **Unclear** | **Unclear** | **Low** |
| **116** | Schwaederlé_2017 | **Unclear** | **Unclear** | **Unclear** | **Low** |
| **117** | Xu_2016 | **Unclear** | **Unclear** | **Unclear** | **Low** |
| **124** | Wang_2020 | **Low** | **Low** | **Low** | **Low** |
| **127** | Jatkoe_2022 | **Low** | **Low** | **Unclear** | **Unclear** |
| **130** | Prabhash_2022 | **Low** | **Low** | **Unclear** | **Low** |
| **131** | Williamson_2022 | **Unclear** | **Unclear** | **Unclear** | **Low** |
| **133** | McKeage_2020 | **Low** | **High** | **Low** | **Unclear** |
| **137** | Iwama_2018 | **Low** | **Unclear** | **Unclear** | **High** |
| **139** | De Luca_2018 | **Low** | **Unclear** | **Unclear** | **Unclear** |
| **140** | Liu_2018 | **Low** | **Low** | **Low** | **Unclear** |
| **147** | De Biase_2013 | **Low** | **Unclear** | **Unclear** | **Unclear** |
| **161** | Mondelo-Macía_2023 | **Unclear** | **Unclear** | **Unclear** | **High** |
| **163** | Sim_2018 | **Unclear** | **Low** | **Low** | **Unclear** |
| **169** | Yao_2017 | **Unclear** | **Low** | **Unclear** | **Low** |
| **172** | Fernandes_2021 | **Low** | **Unclear** | **Unclear** | **Low** |
| **190** | Jiao_2021 | **Low** | **Low** | **Low** | **Low** |
| **192** | Toor_2018 | **Low** | **Unclear** | **Unclear** | **High** |
| **193** | Yang_2018 | **Unclear** | **Unclear** | **Unclear** | **Low** |
| **194** | Jin_2018 | **Unclear** | **Unclear** | **Unclear** | **Unclear** |
| **195** | Guo_2016 | **Unclear** | **Low** | **Low** | **Low** |
| **201** | Horn_2019 | **Unclear** | **Unclear** | **Low** | **High** |
| **174** | Chow_2022 | **High** | **High** | **Low** | **High** |

***Supplementary Table 6 – Applicability Assessment***

| **Applicability** | | | | |
| --- | --- | --- | --- | --- |
| Id |  | **PATIENT SELECTION** | **INDEX TEST** | **REFERENCE STANDARD** |
| **1** | Leest_2023 | **Low** | **Low** | **Low** |
| **4** | Murakami_2022 | **Low** | **Low** | **Low** |
| **5** | Sugimoto_2023 | **Low** | **Low** | **Low** |
| **7** | Cui_2022 | **Low** | **Low** | **Low** |
| **9** | Palmero_2021 | **Low** | **Low** | **Low** |
| **13** | Sepúlveda-Hermosilla_2021 | **Low** | **Low** | **Low** |
| **14** | Zhang_2021 | **Low** | **Low** | **Low** |
| **23** | Tran_2019 | **Low** | **Low** | **Low** |
| **29** | Dono_2019 | **Low** | **Low** | **Low** |
| **30** | Lin_2019 | **Low** | **Low** | **Low** |
| **31** | Li_2019 | **Low** | **Low** | **Low** |
| **39** | Malapelle_2017 | **Low** | **Low** | **Low** |
| **40** | Lambros_2017 | **Low** | **Low** | **Low** |
| **48** | Mehta_2020 | **Low** | **Low** | **Low** |
| **50** | Sabari_2019 | **Low** | **Low** | **Low** |
| **58** | Tachon_2019 | **Low** | **Low** | **Low** |
| **62** | Lettig_2019 | **Low** | **Low** | **Low** |
| **65** | Francaviglia_2019 | **Low** | **Low** | **Low** |
| **70** | Müller_2017 | **Low** | **Low** | **Low** |
| **91** | Reckamp_2016 | **Low** | **Low** | **Low** |
| **93** | Paweletz_2016 | **Low** | **Low** | **Low** |
| **96** | Papadopoulou_2019 | **Low** | **Low** | **Low** |
| **97** | Rachiglio_2016 | **Low** | **Low** | **Low** |
| **98** | Wang_2016 | **Low** | **Low** | **Low** |
| **102** | Couraud_2014 | **Low** | **Low** | **Low** |
| **104** | Leighl_2019 | **Low** | **Low** | **Low** |
| **105** | Remon_2019 | **Low** | **Low** | **Low** |
| **106** | Pritchett_2019 | **Low** | **Low** | **Low** |
| **108** | Mehta_2021 | **Low** | **Low** | **Low** |
| **111** | Lam_2021 | **Low** | **Low** | **Low** |
| **112** | Bauml_2022 | **Low** | **Low** | **Low** |
| **113** | Cui_2017 | **Low** | **Low** | **Low** |
| **114** | Veldore_2018 | **Low** | **Low** | **Low** |
| **115** | Park_2021 | **Low** | **Low** | **Low** |
| **116** | Schwaederlé_2017 | **Low** | **Low** | **Low** |
| **117** | Xu_2016 | **Low** | **Low** | **Low** |
| **124** | Wang_2020 | **Low** | **Low** | **Low** |
| **127** | Jatkoe_2022 | **Low** | **Low** | **Low** |
| **130** | Prabhash_2022 | **Low** | **Low** | **Low** |
| **131** | Williamson_2022 | **Low** | **Low** | **Low** |
| **133** | McKeage_2020 | **Low** | **Low** | **Low** |
| **137** | Iwama_2018 | **Low** | **Low** | **Low** |
| **139** | De Luca_2018 | **Low** | **Low** | **Low** |
| **140** | Liu_2018 | **Low** | **Low** | **Low** |
| **147** | De Biase_2013 | **Low** | **Low** | **Low** |
| **161** | Mondelo-Macía_2023 | **Low** | **Low** | **Low** |
| **163** | Sim_2018 | **Low** | **Low** | **Low** |
| **169** | Yao_2017 | **Low** | **Low** | **Low** |
| **172** | Fernandes_2021 | **Low** | **Low** | **Low** |
| **190** | Jiao_2021 | **Low** | **Low** | **Low** |
| **192** | Toor_2018 | **Low** | **Low** | **Low** |
| **193** | Yang_2018 | **Low** | **Low** | **Low** |
| **194** | Jin_2018 | **Low** | **Low** | **Low** |
| **195** | Guo_2016 | **Low** | **Low** | **Low** |
| **201** | Horn_2019 | **Low** | **Low** | **Low** |
| **174** | Chow_2022 | **Low** | **Low** | **Low** |

***Supplementary Figure 1 – Comparison of Valid Results Between Non-NGS and NGS Tests in Tissue Samples***

***
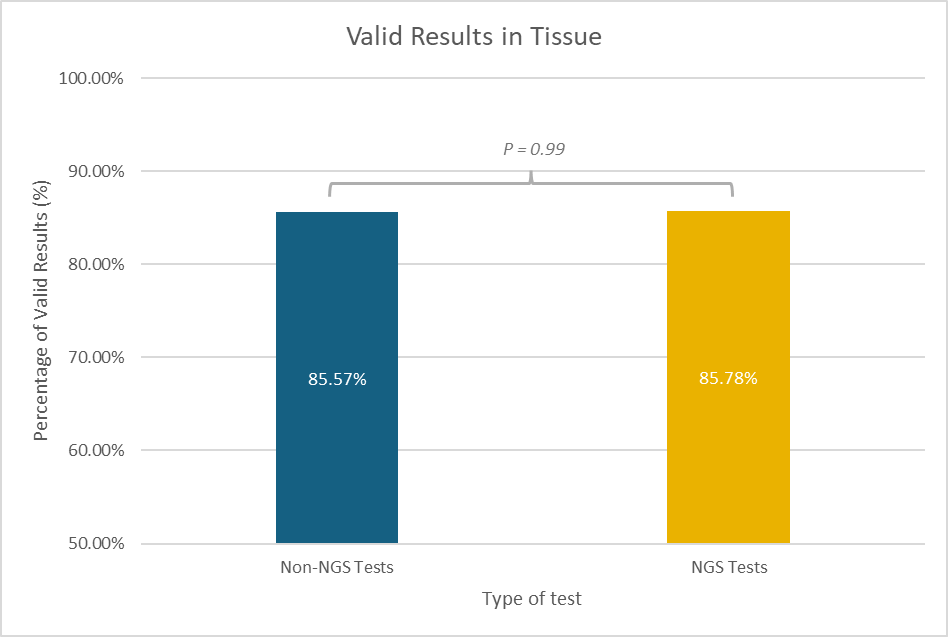
***

*The number of valid results for standard tests in tissue averaged 85.57% (95% CI 59.54–111.60%). For NGS in tissue, the average was 85.78% (95% CI: 62.96–108.60%). No statistically significant difference was found between both methods (p = 0.99).*

***Supplementary Figure 2 – Comparison of Valid Results Between tissue and NGS liquid biopsy***

***
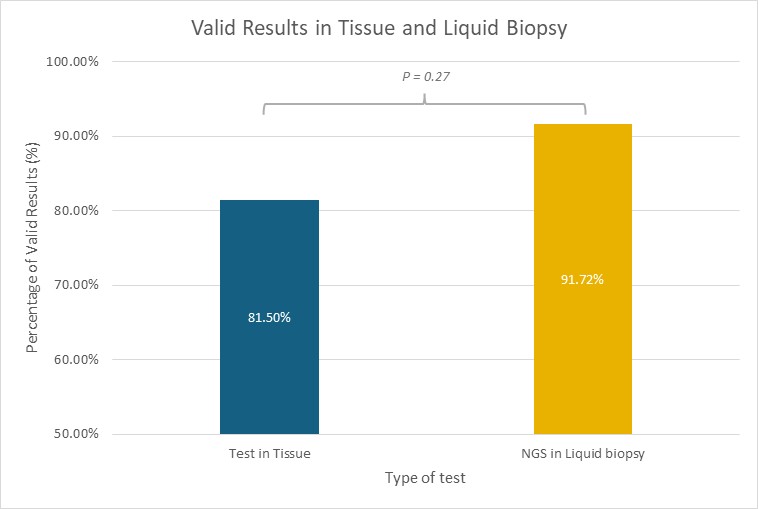
***

*In tissue-based tests, including standard methods and NGS, the percentage of valid results averaged 81.5% (95% CI 65.27–97.73%). For NGS in liquid biopsy, the percentage was 91.72% (95% CI 81.70–101.75%). No statistically significant difference was found between both methods (p = 0.277).*

***Supplementary Figure 3 – Turnaround Between Tests Performed in Tissue Compared to NGS in Liquid Biopsy***

***
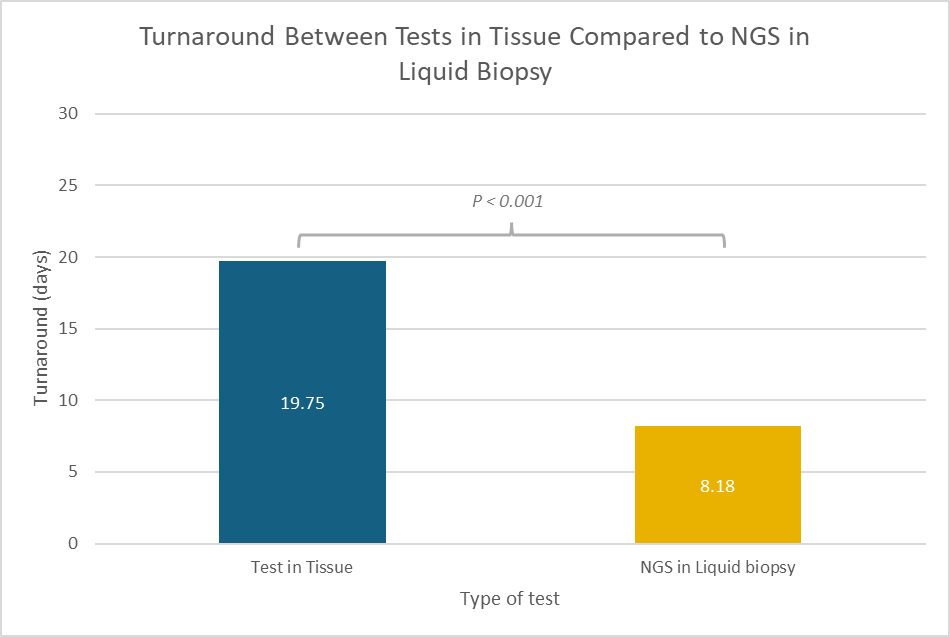
***

*The turnaround time for tests performed in tissue averaged 19.75 days (95% CI 14.49–25 days). In liquid biopsy, the weighted average turnaround time was 8.18 days (95% CI 6.19–10.17 days). The mean difference between both methods was -11.57 days (95% CI -15.41 to -7.73 days), indicating that liquid biopsy provides significantly faster results than tissue testing (p < 0.001).*

***Supplementary Figure 4 – Comparison Between PCR in Tissue and NGS in Tissue for Detection of Mutations in EGFR***


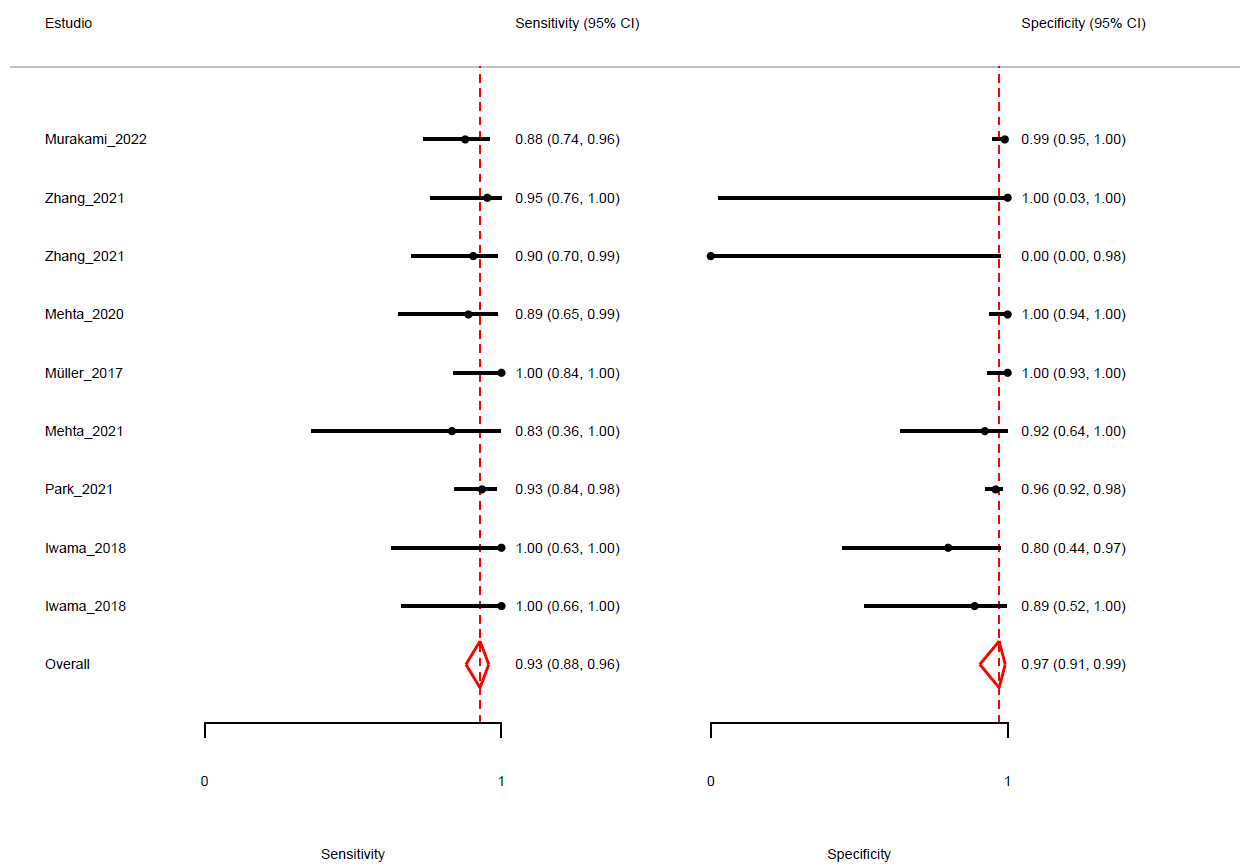


*Epidermal Growth Factor Receptor (EGFR), Next-Generation Sequencing (NGS), Polymerase Chain Reaction (PCR).*

***Supplementary Figure 5 – Comparison Between PCR in Tissue and NGS in Tissue for Detection of rearrangements in ALK***


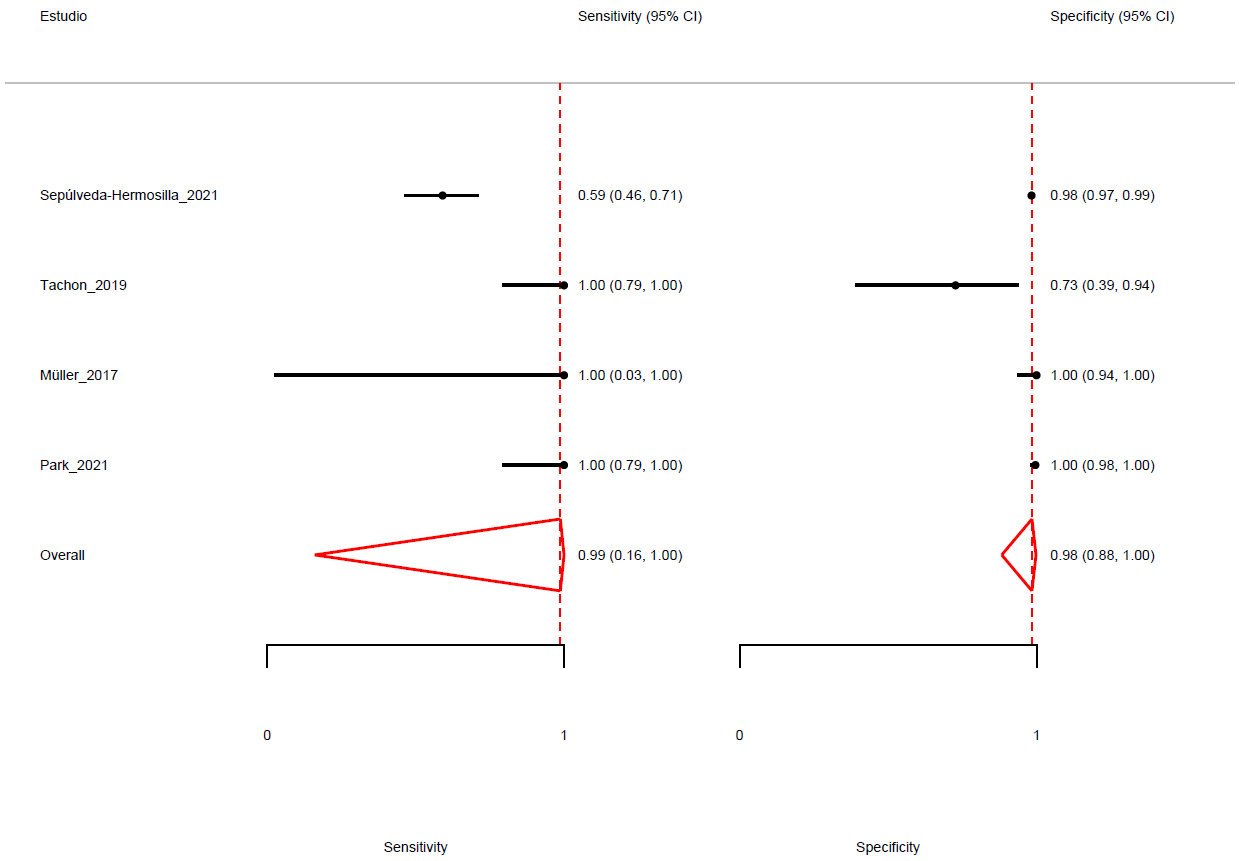


*Anaplastic Lymphoma Kinase (ALK), Next-Generation Sequencing (NGS), Polymerase Chain Reaction (PCR).*

***Supplementary Figure 6 – Performance of NGS in Liquid Biopsy for Detecting alteration of EGFR, mutation KRASG12C, BRAFV600E and alterations of HER2***


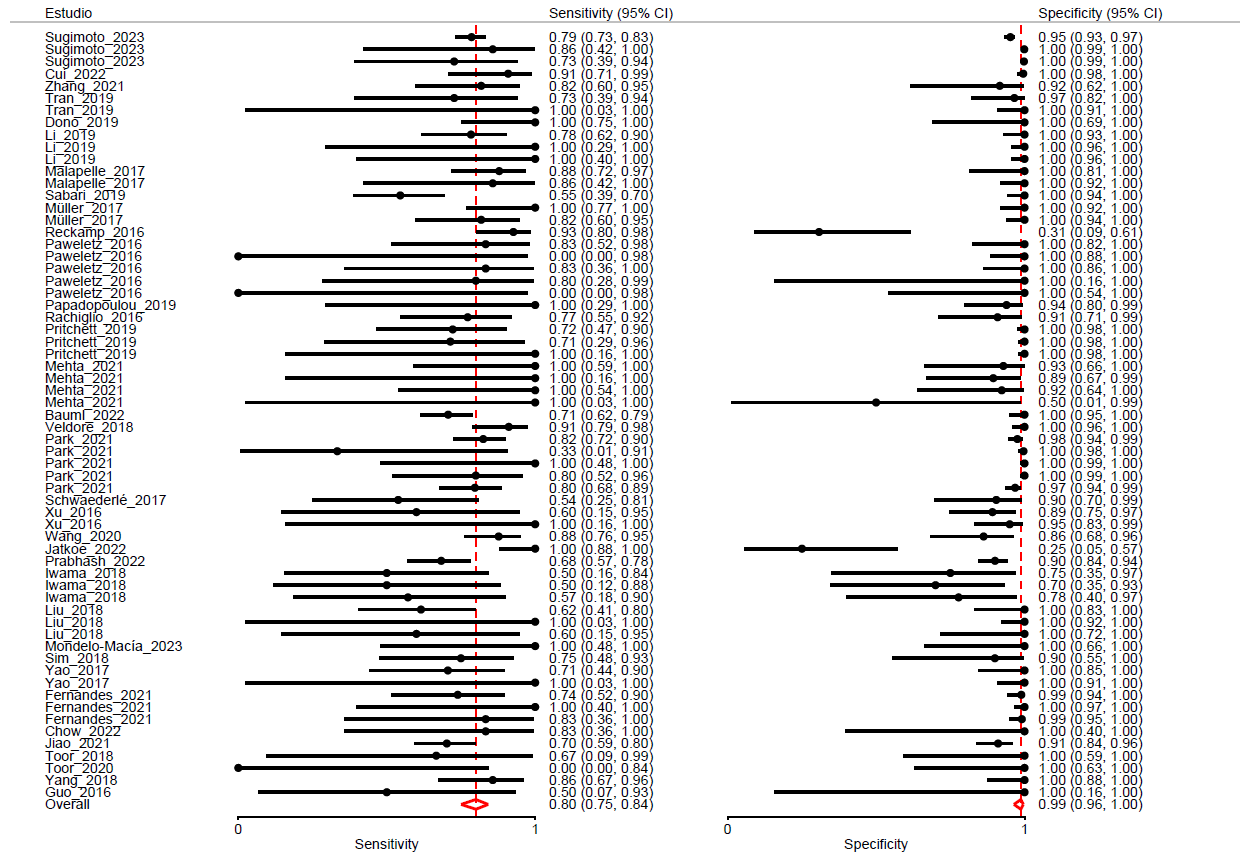


*The forest plot displays the comparisons that evaluated the diagnostic performance of detecting mutations in EGFR, BRAF V600E, KRAS G12C, and HER2 exon 20 mutations. V600E mutation (BRAF V600E), Epidermal Growth Factor Receptor (EGFR), Human Epidermal Growth Factor Receptor 2 (HER2/ERBB2), Kirsten Rat Sarcoma Viral Oncogene Homolog, G12C mutation (KRAS).*

***Supplementary Figure 7 – Performance of NGS in Liquid Biopsy for Detecting Rearrangements of ALK, ROS1, RET and NTRK***

***
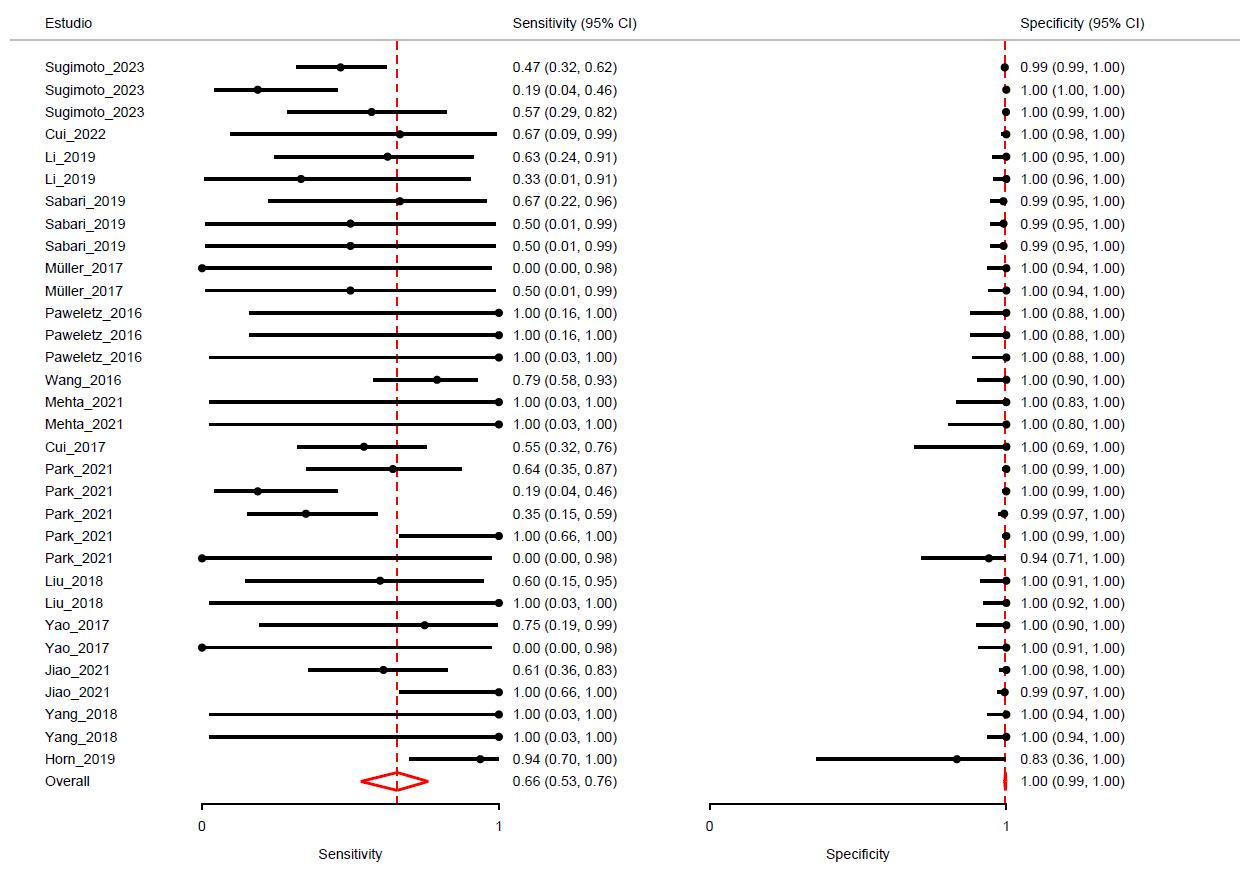
***

*The forest plot shows the comparisons that evaluated the diagnostic performance of detecting rearrangements in ALK, ROS1, RET, and NTRK. Anaplastic Lymphoma Kinase (ALK), B-Raf Proto-Oncogene, Neurotrophic Tyrosine Receptor Kinase (NTRK), Ret Proto-Oncogene (RET), C-Ros Proto-Oncogene 1 (ROS1).*

***Supplementary Figure 8 – Comparison between SOC + NGS in tissue and NGS in liquid biopsy for detecting rearrangements in ALK
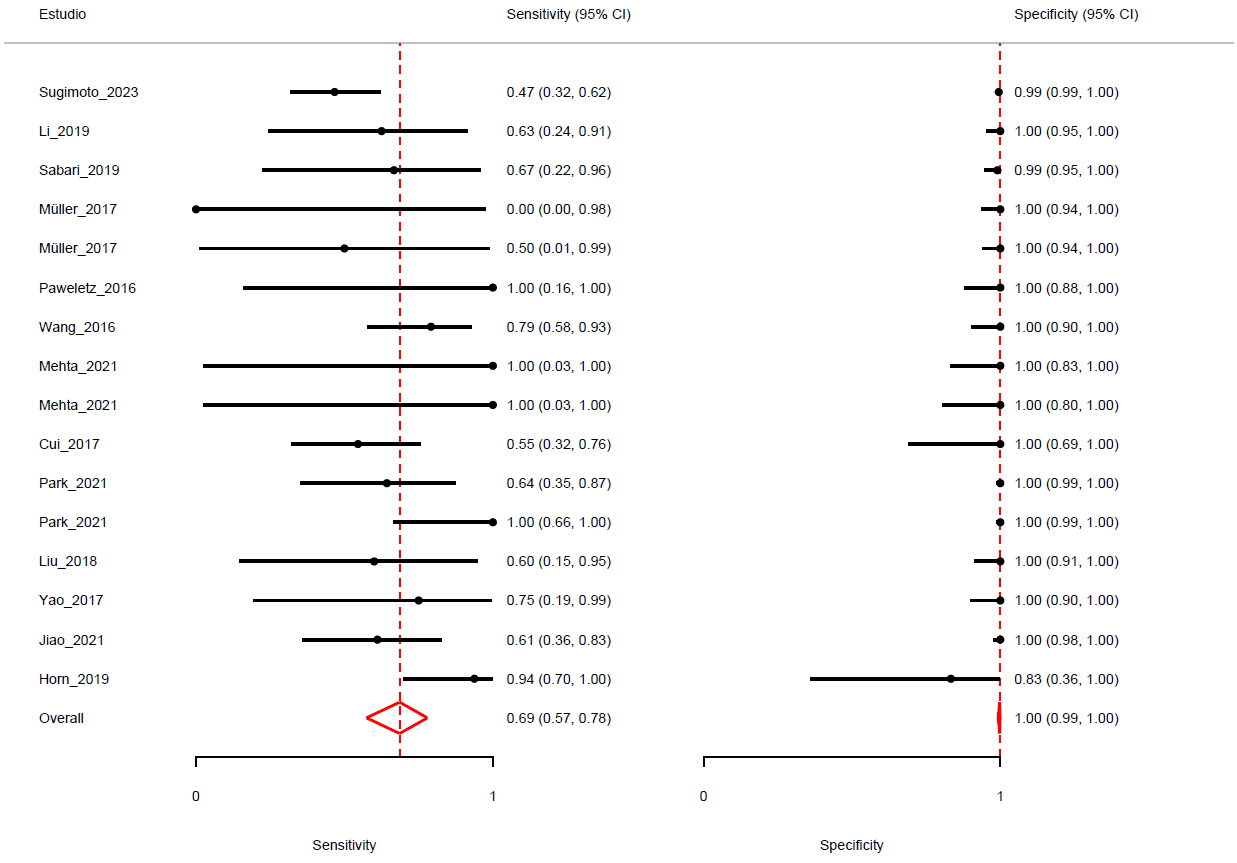
***

*Anaplastic Lymphoma Kinase (ALK), Next-Generation Sequencing (NGS), Standard of Care (SOC).*

***Supplementary Figure 9 – Comparison between SOC + NGS in tissue and NGS in liquid biopsy for detecting BRAF V600E mutation***

***
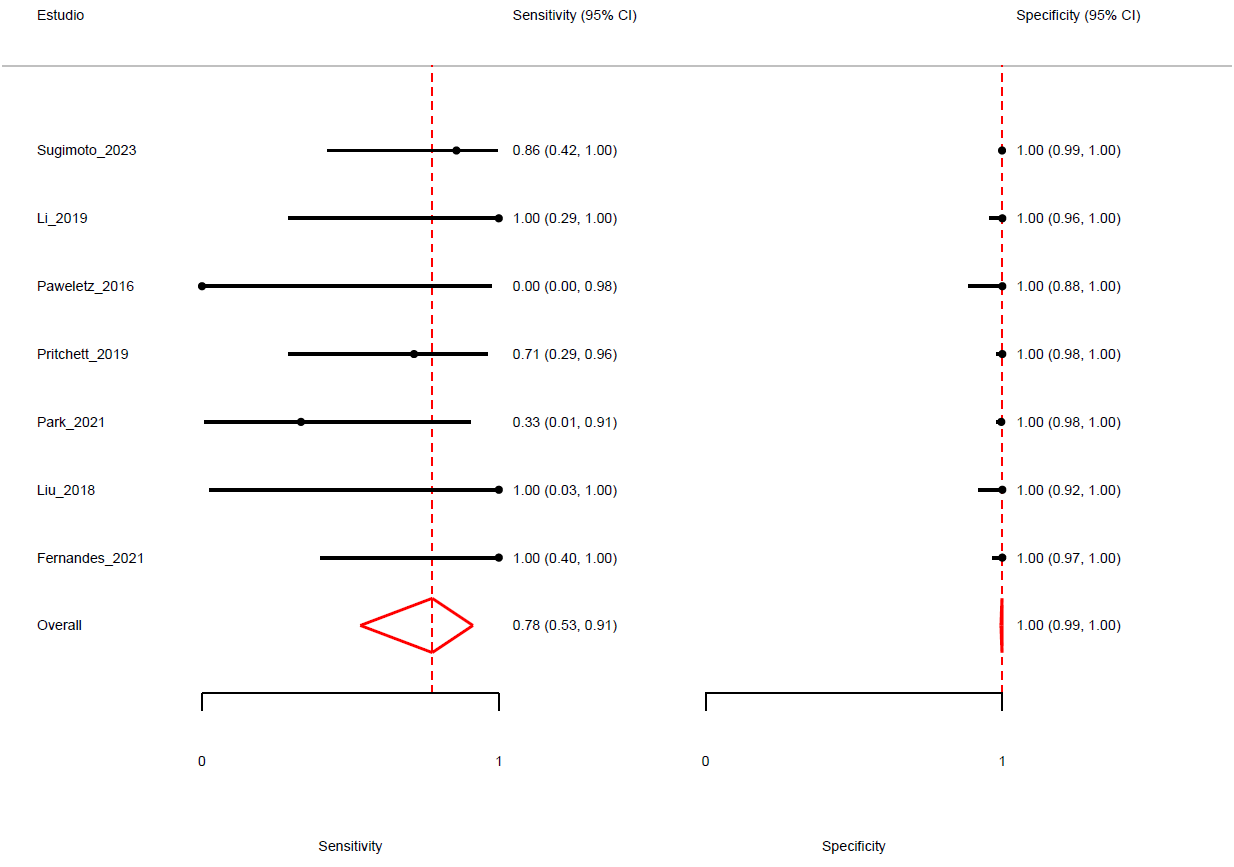
***

*B-Raf Proto-Oncogene, V600E mutation (BRAF V600E), Next-Generation Sequencing (NGS), Standard of Care (SOC).*

***Supplementary Figure 10 – Comparison between SOC + NGS in tissue and NGS in liquid biopsy for detecting EGFR mutations***

***
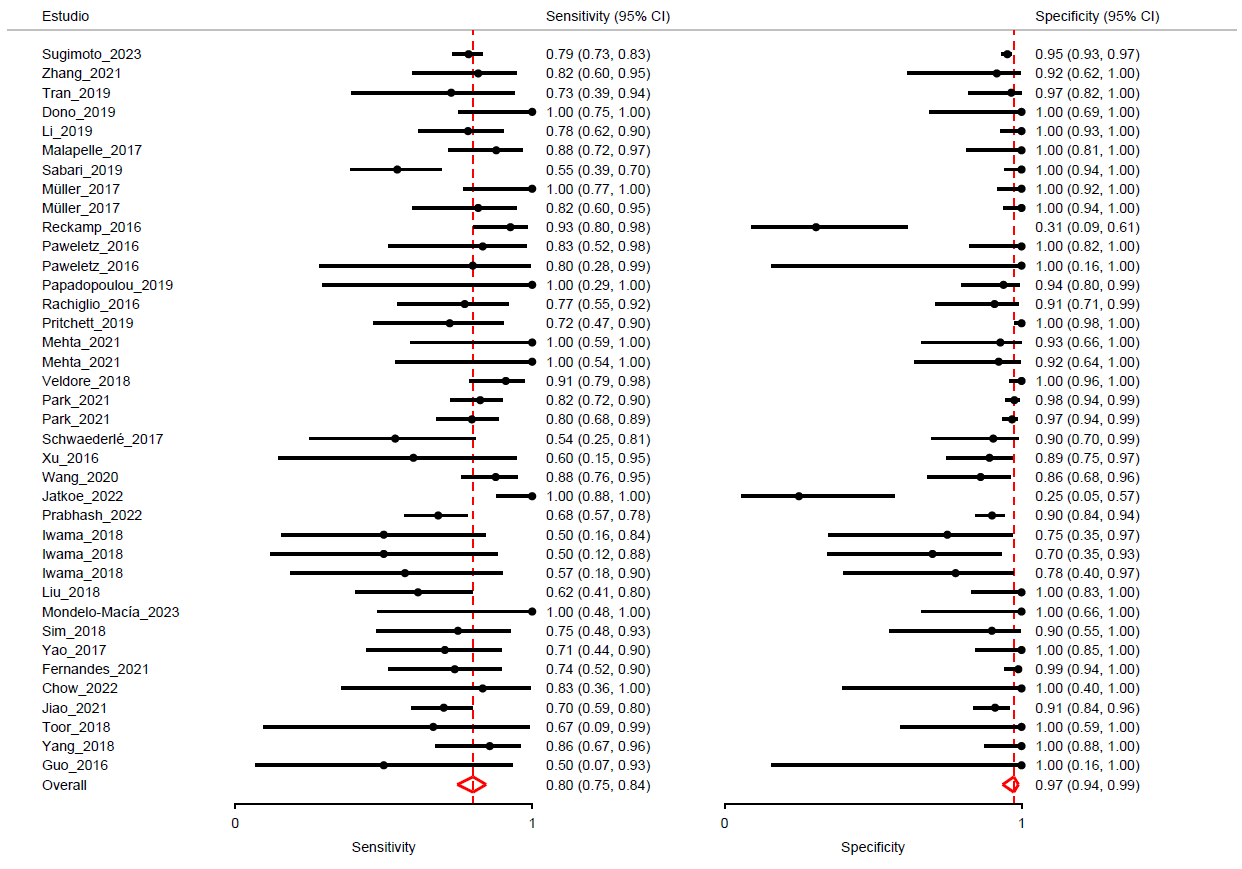
***

*Epidermal Growth Factor Receptor (EGFR), Next-Generation Sequencing (NGS), Standard of Care (SOC).*

***Supplementary Figure 11 – Comparison between SOC + NGS in tissue and NGS in liquid biopsy for detecting KRAS G12C mutation***

***
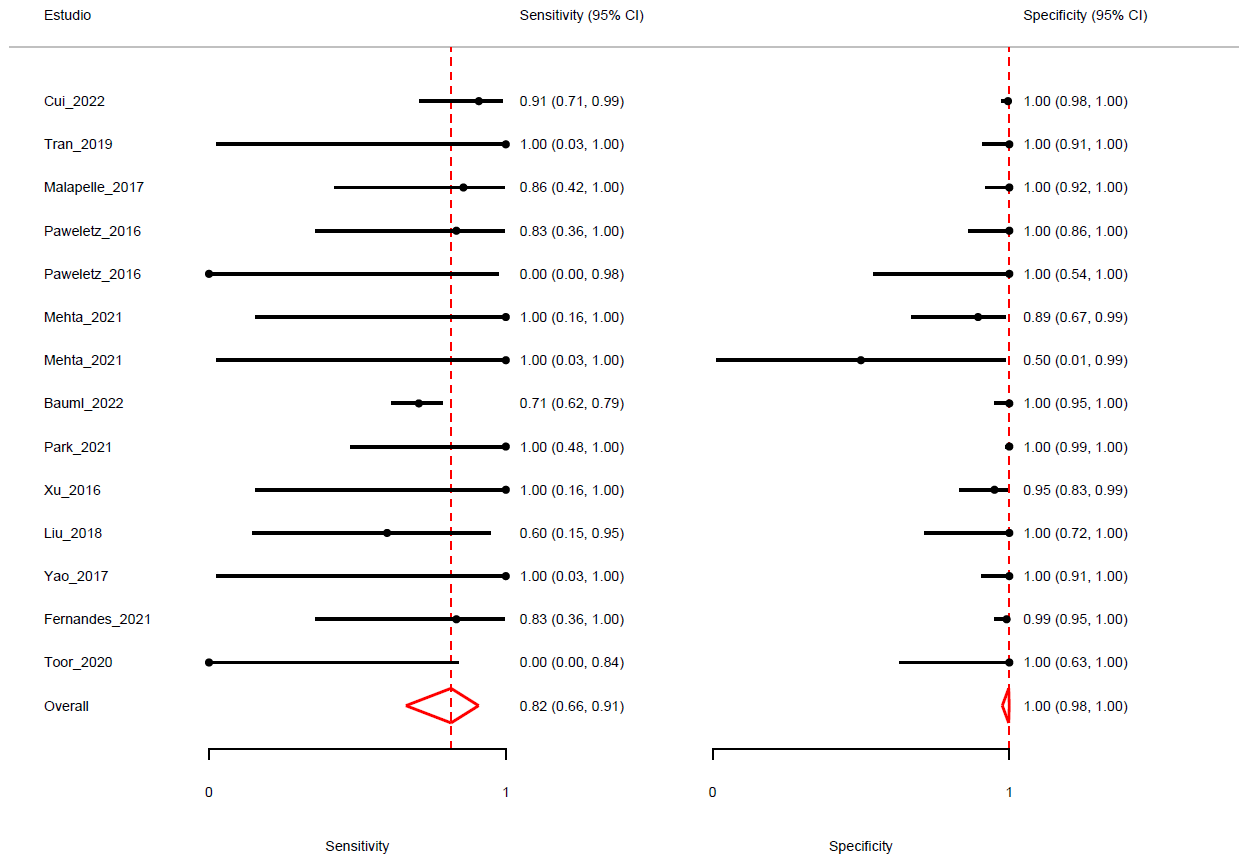
***

*Kirsten Rat Sarcoma Viral Oncogene Homolog G12C mutation (KRAS G12C), Next-Generation Sequencing (NGS), Standard of Care (SOC).*

***Supplementary Figure 12 – Comparison between SOC + NGS in tissue and tissue NGS for detecting MET alterations***

***
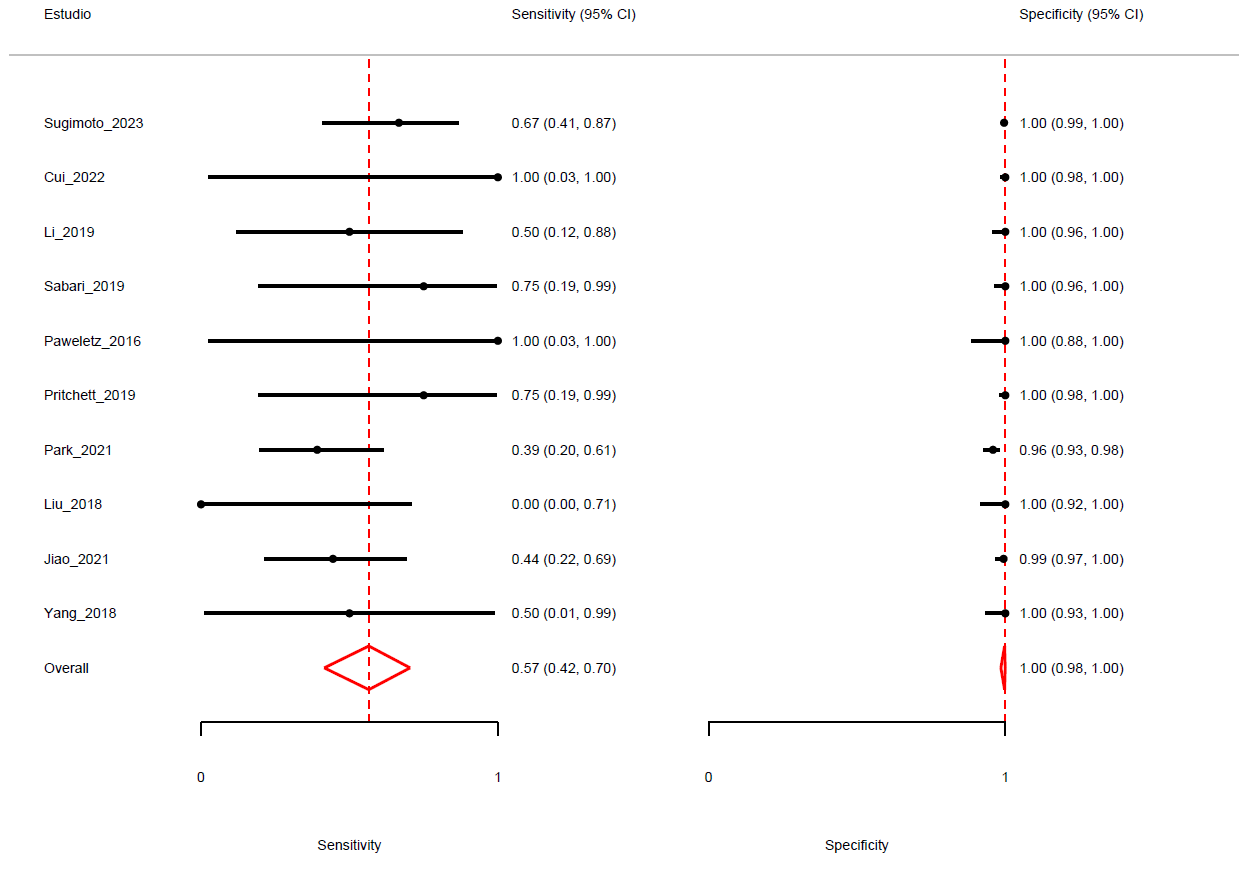
***

*MET Proto-Oncogene (MET), Next-Generation Sequencing (NGS), Standard of Care (SOC).*

***Supplementary Figure 13 – Comparison between SOC + NGS in tissue and NGS in tissue for the detection of RET rearrangements***

***
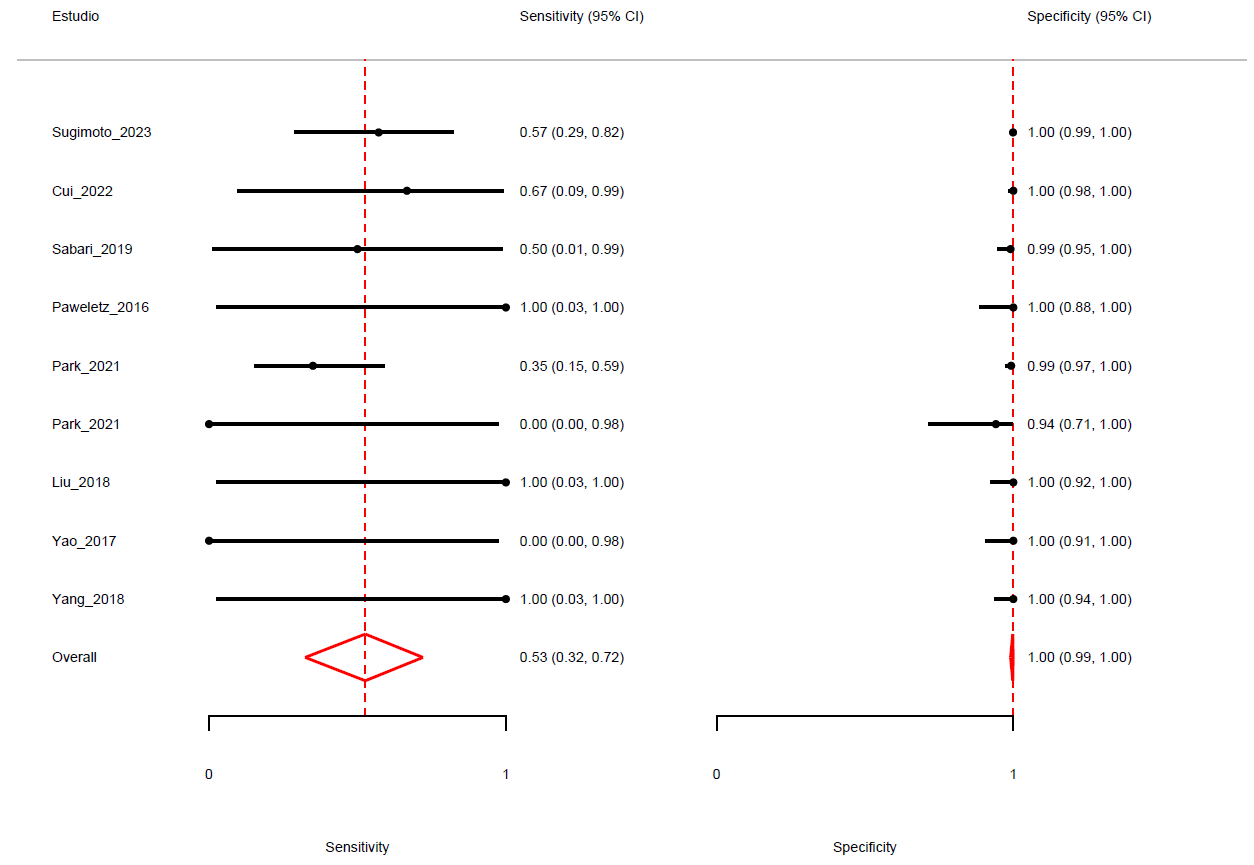
***

*Next-Generation Sequencing (NGS), Ret Proto-Oncogene (RET), Standard of Care (SOC).*

***Supplementary Figure 14 – Comparison between SOC + NGS in tissue and tissue NGS for the detection of ROS1 rearrangements***

***
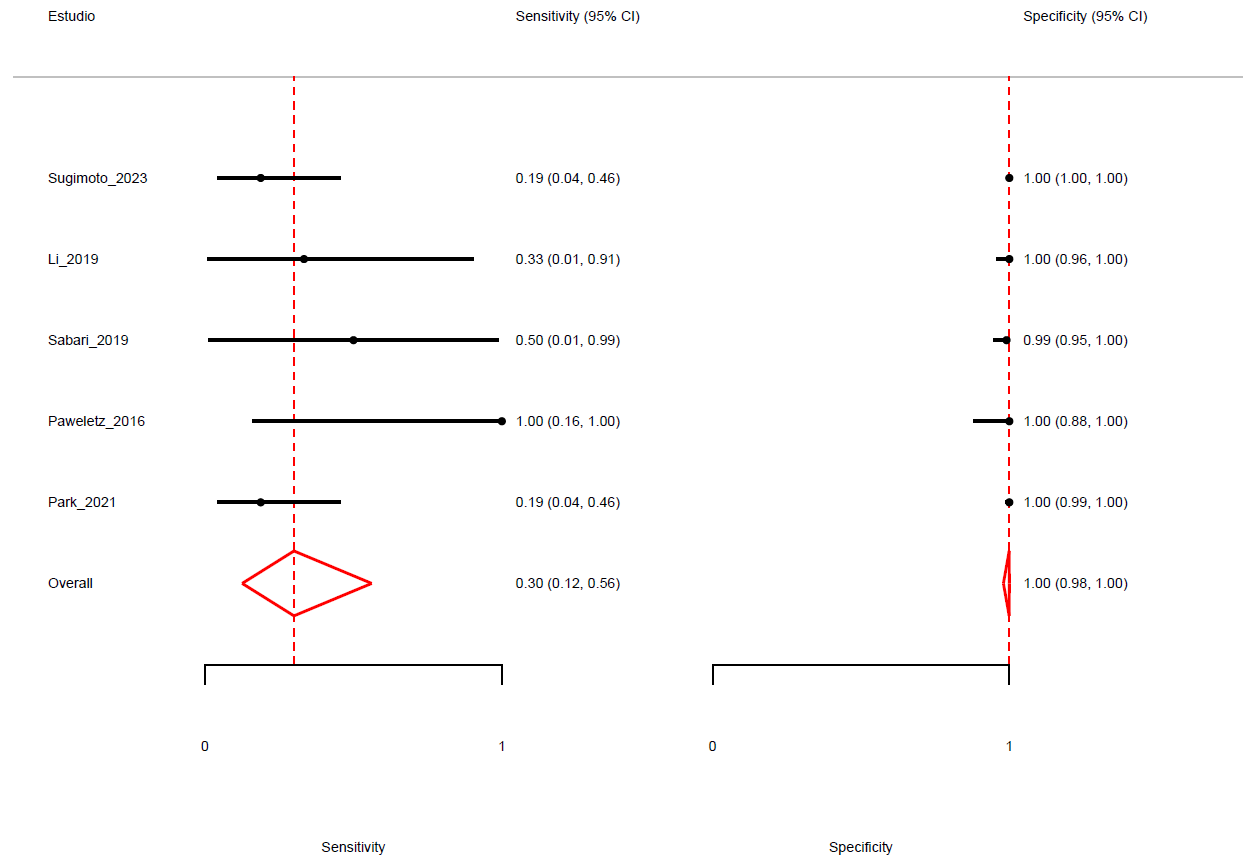
***

*Next-Generation Sequencing (NGS), Standard of Care (SOC), Ret Proto-Oncogene (RET).*
